# P-1903. P4 Pharmacy Students Facilitating Simulated ID Rounds: Perceived Impact on Professional Development

**DOI:** 10.1093/ofid/ofaf695.2072

**Published:** 2026-01-11

**Authors:** Yuman Lee, Nicole Bradley

**Affiliations:** St. John's University, Queens, NY; St. John's University, Queens, NY

## Abstract

**Background:**

ID Rounds is an innovative educational activity that mimics real-world multidisciplinary infectious disease (ID) rounds for 3rd year pharmacy students (P3s) enrolled in an ID elective. Incorporating near-peer teaching, 4th year pharmacy students (P4s) completing their Advanced Pharmacy Practice Experiences (APPEs) serve as facilitators by role-playing key healthcare team members, including the ICU fellow, nurse, microbiologist, and radiologist. P4s progressively reveal clinical information, guiding P3s to critically navigate and manage a dynamic patient case. This study evaluated the impact of ID Rounds on P4s' achievement of Curricular Outcomes and Entrustable Professional Activities (COEPA) as outlined by the American Association of Colleges of Pharmacy.

**Methods:**

An anonymous electronic survey was distributed to P4 participants during the 2023–2025 academic years. The survey assessed the perceived impact of ID Rounds based on COEPA and gathered perceptions of the activity using a Likert scale.

**Results:**

All eleven P4s agreed or strongly agreed that facilitating ID Rounds improved their ability to collect pertinent patient information (100%) and analyze clinical information (91%). All indicated improved skills in establishing patient-centered goals, developing and implementing collaborative care plans, and follow-up and monitoring. The activity provided collaborative interprofessional team experience, improving P4s’ ability to identify at-risk patients (72.7%), prevent adverse drug events/medication errors (81.9%), improve medication use (91%), and increased confidence in educating colleagues and applying evidence-based information (91%). Participation encouraged self-reflection and professional growth (91%). All P4s enjoyed the experience, felt it prepared students for APPEs and real-world practice, and expressed interest in facilitating more simulation activities.

**Conclusion:**

Facilitating ID Rounds provided P4s with valuable opportunities to reinforce key Doctor of Pharmacy competencies, including patient care, interprofessional collaboration, and evidence-based decision-making. These findings support near-peer-facilitated simulations as an effective strategy to enhance APPE learning and professional growth.

**Disclosures:**

All Authors: No reported disclosures

